# Composite Materials Based on Hemp and Flax for Low-Energy Buildings

**DOI:** 10.3390/ma10050510

**Published:** 2017-05-07

**Authors:** Przemysław Brzyski, Danuta Barnat-Hunek, Zbigniew Suchorab, Grzegorz Łagód

**Affiliations:** 1 Faculty of Civil Engineering and Architecture, Lublin University of Technology, 40 Nadbystrzycka Str., 20-618 Lublin, Poland; p.brzyski@pollub.pl (P.B.); d.barnat-hunek@pollub.pl (D.B.-H.); 2 Faculty of Environmental Engineering, Lublin University of Technology, 40B Nadbystrzycka Str., 20-618 Lublin, Poland; g.lagod@pollub.pl

**Keywords:** hemp shives, flax straw, thermal conductivity, low-energy buildings, natural composites

## Abstract

The article presents the results obtained in the course of a study on prospective application of flax/hemp wastes as a filling material of lime-based composites in the construction of low-energy buildings. The utilized filler comprised the hydrated lime with clay and Portland cement used as additives. The analysis involved evaluation of such properties as porosity, density, thermal conductivity, absorptivity, permeability, as well as compressive and flexural strength. Depending on the quantity of the filler, the properties of the composite changed. This, in turn, enabled to evaluate whether the utilized composite met the thermal requirements established for low-energy buildings. Afterwards, the obtained data were cross-referenced with the results gathered in the case of a room built of autoclaved aerated concrete. In order to prevent reaching the critical surface humidity, the internal surface temperature had to be calculated. Moreover, the chances of interstitial condensation occurring in the wall made of the analyzed lime–flax–hemp composite were determined as well. The study showed that the composite exhibits low strength, low density, low thermal conductivity, and high absorptivity. The external walls made of the lime–flax–hemp composite receive a limited exposure to condensation, but not significant enough to constitute any threat. The requirements established for low-energy buildings can be met by using the analyzed composite.

## 1. Introduction

One of the basic principles of sustainable development mandates that the impact of the construction sector on the environment should be decreased [1,2,3,4]. In order to achieve that aim, it is necessary to employ environmentally-friendly solutions, such as the materials derived from plants that absorbed CO_2_ throughout their growth period [5,6,7]. These materials are capable of mitigating the environmental impact of other components, such as Portland cement, the manufacturing process of which involves substantial CO_2_ emissions. Employing thermal insulation materials reduces the heating demand, thus decreasing the ecological footprint of the building in the course of its operation. Thermal insulation boards can be manufactured using materials derived from such plants as flax, hemp [8], or sunflower [9]. Utilizing these plants for the production of loose fibers requires no additional processing. Materials of plant origin (including flax straw, hemp and cereal) enjoy increasing popularity as composite filler or timber frame filling [10,11,12,13]. Filling walls with plant materials is usually sufficient to provide adequate thermal insulation. Natural binders, including lime [14] or clay [15] are often used in conjunction with the filling materials. The obtained composite is used for building walls or insulating roof sand floors.

Organic filling materials, such as hemp shives, are recently enjoying an increasing popularity. These shives are obtained from fibrous hemps. Although they contain psychoactive THC, its dry weight does not exceed 0.2% and therefore may be legally cultivated in Poland.

Hemp shives mixed with lime were used for the first time at the turn of 1980s/1990s in France when old timber-framed buildings were being renovated. The composite was employed for the purpose of filling holes in wicker and straw walls. Hemp is an environmentally-friendly material, which absorbs great amounts of CO_2_ throughout its growth period. Tests showed that 370–394 MJ are required for the production of 1 m^2^ of 26 cm-thick composite wall. Over 100 years of material life, it is capable of accumulating 14–35 kg of CO_2_ [16]. Additionally, data from other literature suggests that 1 m^3^ of a lime–hemp composite wall could absorb approximately 110 kg of CO_2_ [10]. Hemp shives in the amount of 1000 kg could absorb an equivalent of about 1800 kg of CO_2_. Approximately 40 m^3^ of lime–hemp composite is required to build a typical house, which in turn is produced from roughly 7–10 tons of hemp shives. This amount is equivalent to a yield from 1 ha of fibrous hemp plantation [17].

Lime – hemp composite serves as filling in timber frames, mainly in the external barriers, due to its adequate insulating parameters. It can be used both as insulation or construction material, depending on which of the two components is used in greater amount. Moreover, the composite can be used as filling for wooden external walls, as well as thermal insulation of roofs and acoustic insulation of floors. Although the composite has not been designed as a load bearing structure, if proper binder is used, it may achieve the compressive strengths which are similar to the ones characterizing low class cellular concrete [18]. Unfortunately, higher compressive strength, which results in greater density, simultaneously lowers the thermal insulating properties of the composite [19,20]. Additionally, this material can be used to regulate the level of humidity in a house, thus improving the comfort in relation to houses with ordinary walls [21].

Prospective application of hemp and flax semi-products in construction of buildings has fuelled a growing interest in cultivation of these plants in numerous countries around the world, including Poland. In the late 1960s, the Institute of Natural Fibers and Herbs in Poznan managed to adapt a variety of fibrous hemp to Polish climatic and soil conditions. This variety was subsequently patented and called “Białobrzeskie”. “Białobrzeskie” hemp is a tall (3–4 m) plant, characterized by low THC content (<0.2%) and a high growth of fiber. These factors contributed to its popularity and “Białobrzeskie” is now the most common variety of hemp cultivated on Polish plantations.

Cultivation of hemps has the added environmental benefit of being herbicide-free. These plants are highly resistant to both diseases and insects. Moreover, the spent material made from hemp can be cheaply recycled. As far as agriculture is concerned, hemps constitute essential herbicides and repellents. Hemps can grow to be 0.5 m to 4 m tall, depending whether the climate and soil are favorable or not. Fibrous hemps prefer a pH close to 7. Approximately 11.4 MJ of energy is required to cultivate hemp on a 1 ha field. By contrast, cultivating wheat would require about 18.1 MJ, while corn would need 23 MJ [22]. 

The paper presents the results of studies on the application of local flax and hemp wastes in construction of buildings, mainly in the external barriers. Both the mechanical, as well as physical properties of lime–hemp and lime–flax–hemp composites were analyzed. The study presents the results of a test involving 12 composite recipes, differing in respect to the type of the utilized binder and filler, as well as their ratio. Additionally, the possibility of moisture condensation was calculated. This issue is especially valid for the organic origin materials prone to microbiological contamination caused by water presence. Water present in building materials may decrease their thermal and mechanical properties [23,24,25]. However, it mainly impacts the indoor air quality and leads to Sick Building Syndrome phenomenon [26,27,28].

## 2. Materials and Methods

### 2.1 Manufacturing of the Composite Materials

**Figure 1:**
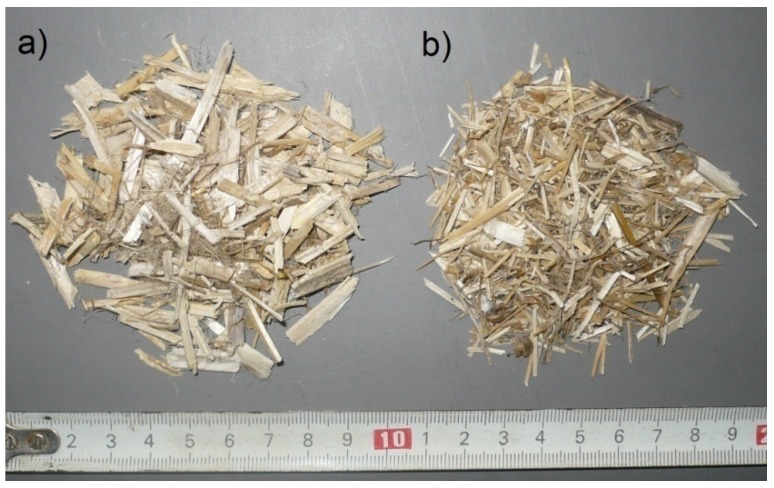
Fillers used for composites: (**a**) hemp shives; and (**b**) flax straw.

**Figure 2:**
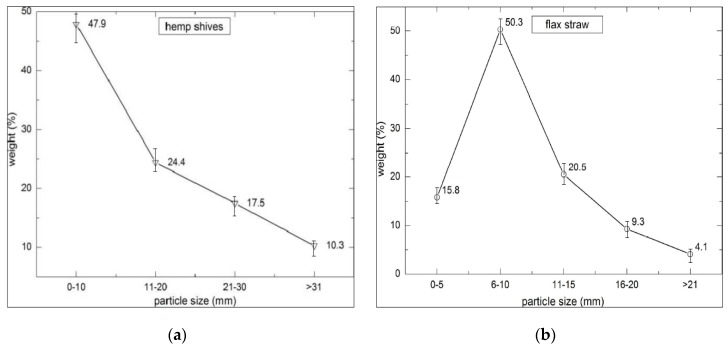
Fraction of: (**a**) hemp shives; and (**b**) flax straw in composites (error bars mean standard deviation of particular fraction mass).

The tests were conducted in two series that differed in respect to filler composition. Hemp shives of “Białobrzeskie” variety, adapted to Polish climate conditions and soil (Institute of Natural Fibers and Herbs, Poznan, Poland) were applied as an organic filler for Series 1 (H1–H6) (Figure 1a) as well as sand, in lower quantity. On the other hand, hemp shives enriched with flax straw (Institute of Natural Fibers and Herbs, Poznan, Poland) were employed for Series 2 (FH1–FH6); see Figure 1b. The obtained fillers exhibit low density (roughly 100 kg/m^3^), high porosity (approximately 80%), and high weight absorptivity (reaching roughly 400% of dry weight following 48 hours of soaking). The length, width, and fraction thickness of the utilized hemp shives and flax straw were greatly diversified. The stalks of flax are different from the ones of hemp, because their structure resembles the stalks of a cereal plant. However, they contain fibers instead. In contrast, hemp stalks constitute fiber-covered woody cores. Flax shives weight content in relation to fraction is presented in Figure 2b.

Each series consisted of six recipes prepared in the laboratory, which were characterized by different quantities of utilized ingredients. The main ingredients of the binder included hydrated lime and Portland cement. Additionally, clay was used as an extra binder in Series 2. Portland cement was used in the amount of 9.5–20.1% by weight of binder in Series 1 and 23% by weight of binder in Series 2. As an exception, the cement mass share in the FH4 recipe, constituted 45.6% of binder composition. In other studies reported in the literature [29], cement was applied in the amount of 2.7–10% by weight in relation to the whole binder. On the other hand, Kinnane at al. [30] applied 10% of cement by weight in relation to all binder compounds.

Table 1 shows the percentage composition of the tested composites.

Hydrated lime of highest possible purity, namely class and largest specific surface was applied for the manufacturing of composites. CL 90-S lime was employed in the research, meaning that Ca(OH)_2_ constitutes at least 90% of its chemical composition. On the other hand, its specific surface amounted to approximately 15,000 cm^2^/g, according to the producer (Lhoist, Górażdże, Poland). The larger is the specific surface, the greater is the contact area of lime with air. This, in turn, translates to faster carbonation.

Mixtures were prepared with CEM I 42.5R Portland cement (Cemex, Chełm, Poland), which is guaranteed to reach the minimum strength of 42.5 MPa after 28 days. Portland cement was added in order to improve the early strength of the composite. This parameter is crucial, as the composite was to be employed in monolith walls.

Figure 2 shows graphs corresponding to the weight content in relation to length of both hemp shives and flax straw. A mix of shives included small amounts of hemp fibers and dust as well.

The length of the flax straws is more homogenous comparing to the hemp shives. According to the literature reports [31], selecting appropriate shives fractions enables to modify certain properties of the lime–hemp composite, for example its durability or thermal conductivity.

This variety of shives mixture was also used in other studies [32]. The prevalent fraction is characterized by the length of 14.8–25.1 mm, thickness of 1.8–3.1 mm and width of 2.8–5.4 mm.

Water constituted the last ingredient of the mixture. It is vital to correctly determine the quantity of water in composite organic fillers. Only a bare minimum, necessary for the binder to harden should be applied, because too much water will lengthen the time required for drying of composite walls. High absorptivity of hemp and flax shives should be taken into consideration.

### 2.2. Preparation of Mixture and Specimen

There is no single method of mixing the ingredients, reported in the literature. Two different approaches to the problem of mixing the ingredients can be distinguished. The first one was presented by Cerezo [33] and Nguyen [34] in which the hemp shives were first submerged in water, in order to subsequently add the soaked shives to binder materials. On the other hand, Hirst et al. [35] and Gourlay et al. [36] took a different approach and simply added dry hemps shives to the liquid binder.

Mixtures applied for the tested composites were obtained by mixing the binding materials together with water, and gradually adding the resulting liquid binder to the pre-mixed filler components. Mixing lasted until uniform, paste-like consistency was achieved.

The obtained mixtures were placed in molds. However, taking into account their inconvenient shape and low weight, hemp and flax shives could not be compacted in the molds under gravity. Instead, they had to be compacted manually in two layers of 75 mm thickness with a wooden rod (diameter of 30 mm). A similar procedure was described in the literature reports, where 50 mm-thick layers were mechanically compacted by means of a press operating under the stress of 0.05 MPa [37]. It is also possible to compact the mixture in molds by hand, without resorting to any tools, as shown in the literature [38].

After mixing, the samples were left in molds to mature. After two days, the molds were removed, but the obtained composite was still ductile. The material has not hardened owing to high water content and air binder quantity. Maturation of specimens lasted for 28 days (including 2 days in molds after formation) in air-dry conditions at 20 ± 2 °C and a relative humidity equaling 60 ± 5%. 

Separate set of three samples from each of the series was prepared for each test.

### 2.3. Methods

#### 2.3.1. Determination of Physical and Mechanical Properties of the Composites

The properties of specimens were determined following 28 days of maturation. The tests of apparent density were carried out in line with EN 12390-7:2001 standard, whereas the absorptivity tests were conducted according to EN 13755:2008 standard 100 × 100 × 100 mm^3^ (cubic) specimens. The samples were dipped into the water entirely. The absorptivity test involved increasing the weight of water-soaked specimens until total saturation was achieved. The result was the ratio between the mass of absorbed water and the mass of dry sample. The samples remained submerged for roughly 7 days.

The porosity of samples was specified on the basis of the density, which was determined with pycnometric method beforehand, in line with EN 1936:2010 standard.

**Figure 3:**
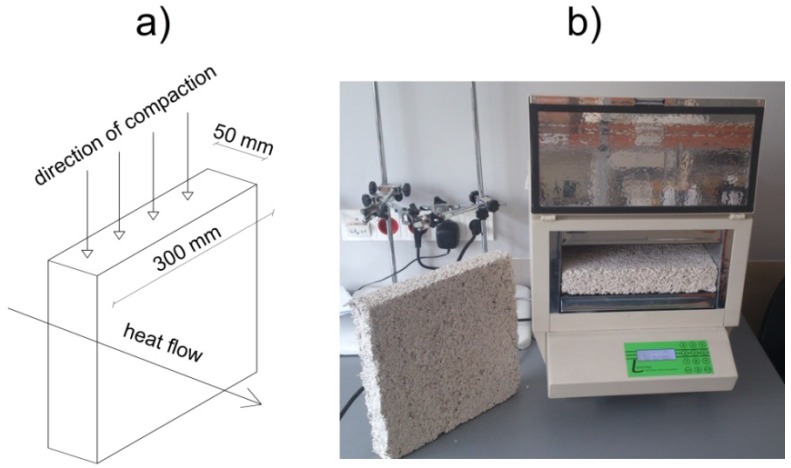
Direction of molding specimens to thermal conductivity test (**a**); and measuring stand (**b**).

Testing the thermal conductivity was performed with a FOX 314 (TA Instruments, New Castle, DE, USA) plate apparatus. It involved samples dried to a constant weight and having the dimensions of 300 × 300 × 50 mm^3^. The temperature of the heating and cooling plate amounted to 25 °C and 0 °C, respectively, and the average temperature was equal to 12.5 °C. The method of specimen preparation prior to the thermal conductivity test, as well as the measuring stand, are both presented in Figure 3. Thermal conductivity of the material was examined according to its position in a real building barrier, considering the direction of heat flow, composition positioning and compacting in the formworks. The composition was placed in molds and compacted vertically. During the measurement, the direction of heat flow between the heating and cooling plate was perpendicular to the direction of compaction, similarly as in the real object.

Two recipes of composites with the lowest λ value from both series (one recipe from series 1 and one recipe from series 2) were tested for water vapor permeability coefficient (δ) using dry-cup method—in line with the EN 12086 standard. The samples were cylindrical in shape, with the dimensions of 90 mm in diameter and 16 mm in height. Additionally, the diffusion resistance factor (μ) was calculated using the obtained water vapor permeability coefficient, by means of the following formula:

(1) $$\mu =\frac{\delta_{0}}{\delta }[-]$$

where *δ_0_*is the water vapor permeability of air, 2 ×10^–10^ kg/(m∙s∙Pa), and δ is the water vapor permeability coefficient of tested material kg/(m∙s∙Pa).

**Figure 4:**
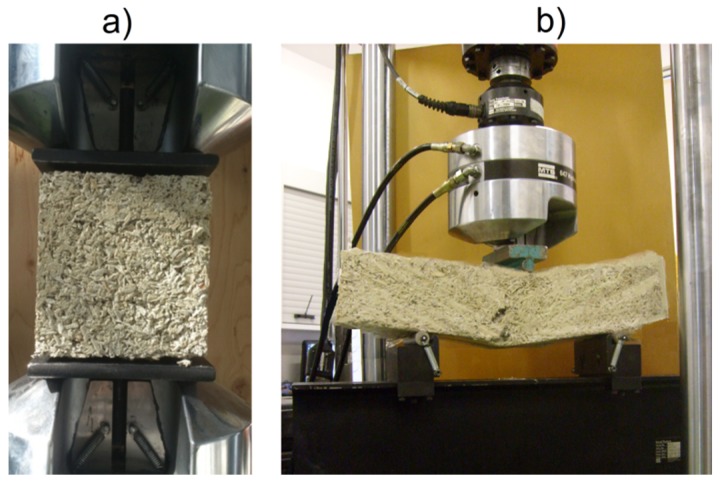
Composite mechanical properties determination: (**a**) compressive strength test, and (**b**) flexural strength test.

Compressive and flexural strength was determined by means of a MTS 810 hydraulic press (MTS System Corporation, Eden Prairie, USA) with a load range of 0–100 kN (Figure 4a).

The literature reports present data concerning determination of compressive strength characterizing the biological composites. Arnaud et al. [14] operated the press with displacements of 5 mm/min. Walker et al. [37] applied the lowest permissible increases in the compressive strength amounting to 50 N/s, according to EN 459-2 standard concerning building lime test methods. Benfratello et al. [39] utilized the displacement of 0.2 mm/min in order to conduct a more detailed curve analysis of materials characterized by low strength. In our research for the compressive strength test, the press head was controlled with 3 mm/min displacements. Three cubic samples of each mixture, characterized by the dimensions of 150 × 150 × 150 mm^3^, were used for this purpose.

As far as the flexural stress is concerned, another three samples of each mixture, having the dimensions of 100 × 100 × 500 mm^3^ were used, in accordance to EN 12390-5:2009 standard (Figure 4b). The samples were placed on supports with 300 mm spacing. Afterwards, a centrally placed force was applied to the specimens (3-point-bending), with the load increasing by 50 N/s.

#### 2.3.2. Water Vapor Condensation 

**Figure 5:**
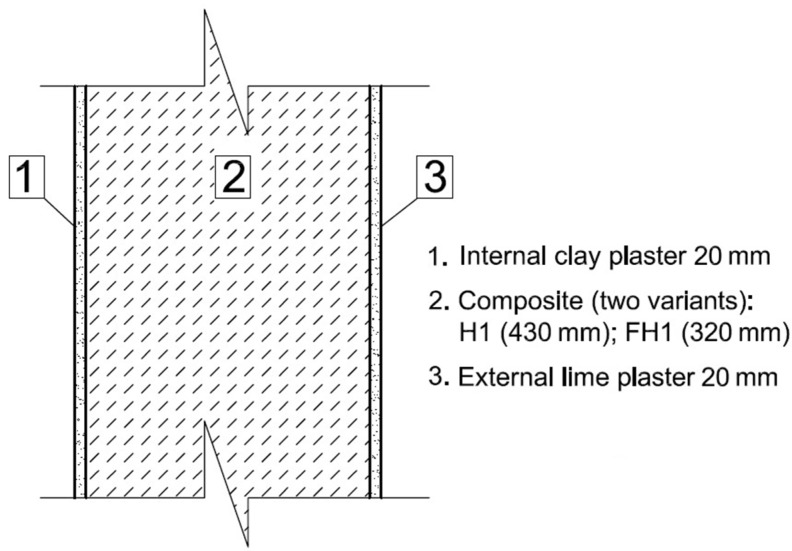
Schematic views of external wall in H1 and FH1 technology.

The partitions comprising organic materials ought to be designed in the manner preventing condensation from occurring. In this section, a technical parameter (water vapor condensation) of an exemplary construction external building partition made of the tested composites with external lime plaster and internal clay plaster is considered. Cross-sections showing the construction of two variants of the external walls made of lime–hemp composite (H1) and lime–flax–hemp composite (FH1) are presented in Figure 5.

Calculation of water vapor condensation was conducted in line with EN 13788 standard. The procedure was divided into two sections. The internal surface temperature necessary to avoid the critical surface humidity was described in the first section, whereas the possibility of interstitial condensation was established in the second one.

In order to conduct calculations, the value of overall heat transfer coefficient U was assumed, calculated according to EN ISO 6946:2008 standard for both analyzed model walls at the level of 0.25 W/(m^2^·K). According to our own investigations and EN ISO 6946:2008 standard, the corresponding thickness of composite layer required to achieve this value equaled 430 mm for wall H1 and 320 mm in the case of wall FH1, which depended on thermal conductivity coefficient of particular composite. Thermal conductivities of the composites were determined experimentally, and thermal conductivities of both plasters were assumed according to EN ISO 6946:1999 standard and [40]. Coefficients of water vapor permeability (δ) of the considered composites were also determined experimentally, whereas the coefficients of water vapor permeability of plaster materials were assumed according to EN-ISO 10456:2003 standard and [40]. A similar analysis concerning water vapor transport in wall construction based on other composite recipe was presented in article [41]. For the calculations, wall section was divided into 18 layers with constant thermal resistance factor R = 0.2 (m^2^·K)/W.

**Figure 6:**
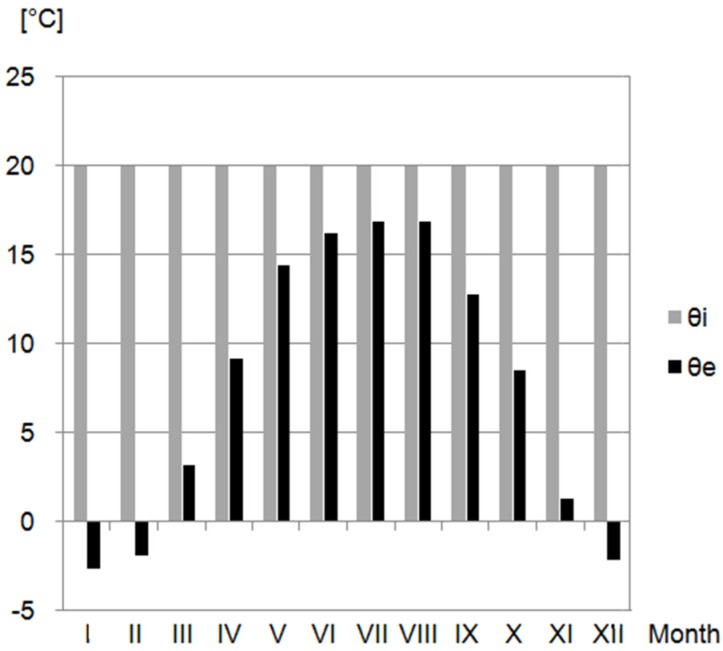
Indoor and outdoor temperature of the air [42].

**Figure 7:**
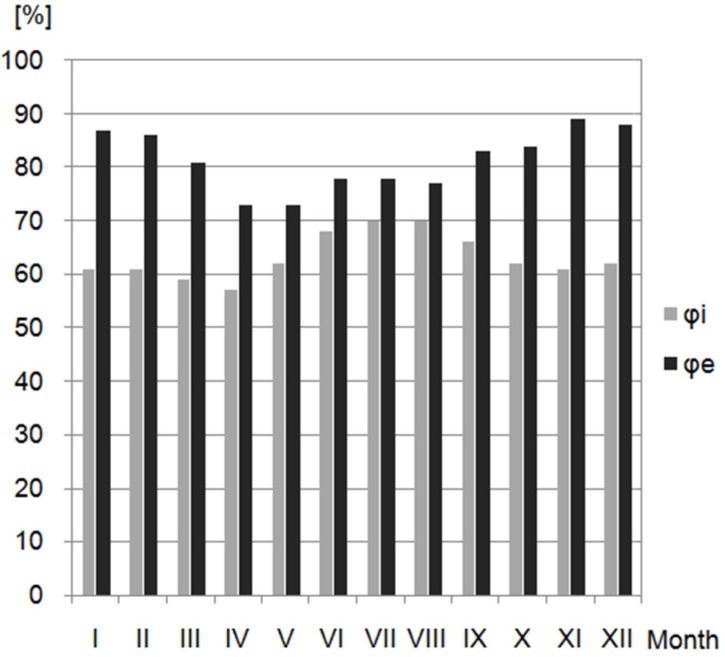
Indoor and outdoor relative humidity of air [42].

The meteorological data adopted for each month were taken from the weather station Lublin-Radawiec, Poland. The obtained data included the average outdoor and indoor temperature (θ_e_, θ_si_), as well as the relative humidity (φ_e_, φ_i_), represented in Figure 6 and Figure 7, respectively.

## 3. Results

### 3.1. Physical Parameters of the Composites

#### 3.1.1. Density and Porosity

**Figure 8:**
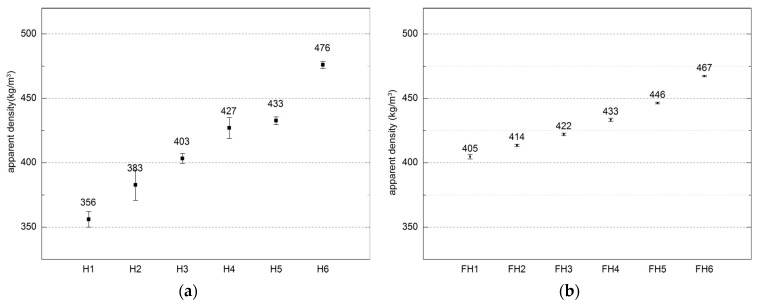
Apparent density of composites (error bars mean standard deviations of apparent density): (**a**) Series 1, (**b**) Series 2.

Figure 8 shows the apparent density of both tested composite series.

**Figure 9:**
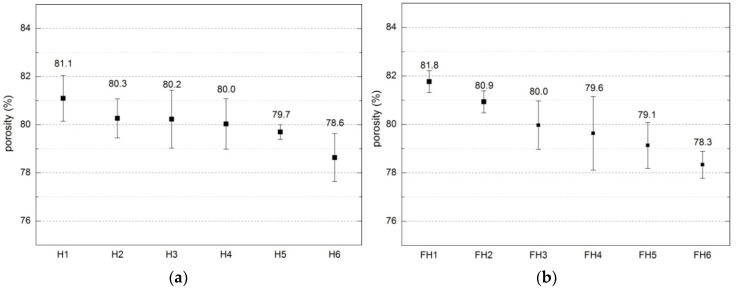
Total porosity of composites (error bars mean standard deviations of porosity): (**a**) Series 1, (**b**) Series 2.

Figure 9 shows the porosity of both analyzed series of the composites.

#### 3.1.2. Composites Absorptivity

**Figure 10:**
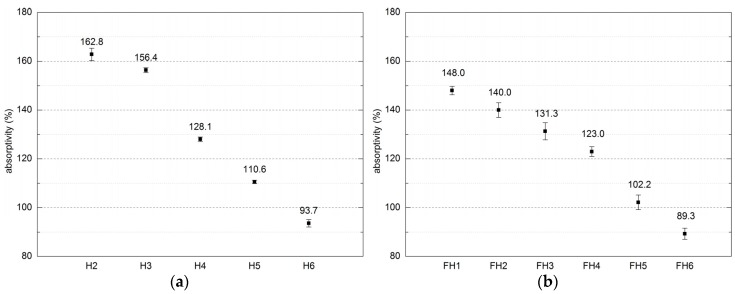
Mass absorptivity of composites (error bars mean standard deviation of absorptivity): (**a**) Series 1, (**b**) Series 2.

The mass absorptivity of both analyzed composite series is presented in Figure 10.

#### 3.1.3. Thermal Conductivity Coefficient

**Figure 11:**
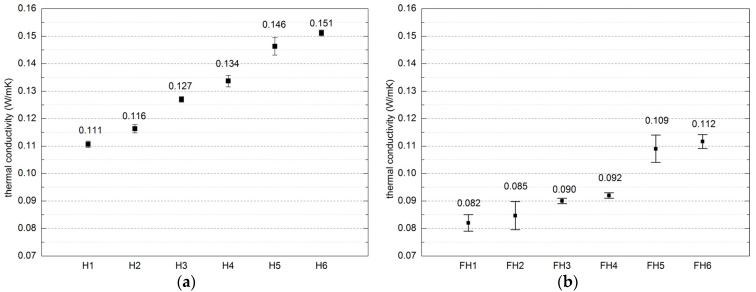
Thermal conductivity coefficient of composites (error bars mean standard deviation of heat conductivity coefficient): (**a**) Series 1, (**b**) Series 2.

Figure 11 presents the results thermal conductivity coefficient values reached for both series of composites.

#### 3.1.4. Water Vapor Permeability Coefficient and Diffusion Resistance Factor

Table 2 shows the average water vapor permeability coefficient δ and diffusion resistance factor µ obtained for H1 from Series 1 and FH1 from Series 2 composites.

### 3.2. Mechanical Properties of the Material

Table 3 presents the calculated mechanical properties of composites.

### 3.3. Calculation of Condensation Process

#### 3.3.1. Internal Surface Temperature to Avoid Critical Surface Humidity

The following data were used for the calculation: thermal conductivities of H1 and FH1 composites (from authors’ own investigations, presented in Figure 11). Thermal conductivities of plaster materials, lime plaster 0.70 W/(m·K) and clay plaster 0.91 W/(m·K), were assumed according to EN ISO 6946:1999 standard and [40].

The results of calculations of the internal surface temperature to avoid critical surface humidity are presented in Table 4. For each month, it was calculated the value of temperature coefficient f_Rsi,min_. Its greatest value is considered as critical f_Rsi,crit_, which in this case corresponds to January.

#### 3.3.2. Determination of the Possibility of Interstitial Condensation

The following data were used for the calculation: coefficients of water vapor permeability (δ) of the composites (from authors’ own investigations, presented in Table 2). Coefficients of water vapor permeability of plaster materials were as follows: lime plaster 2 × 10^−11^ kg/(m∙s∙Pa) in accordance with EN-ISO 10456:2003 standard and [40] clay plaster 2.5 × 10^−11^ kg/(m∙s∙Pa).

**Figure 12:**
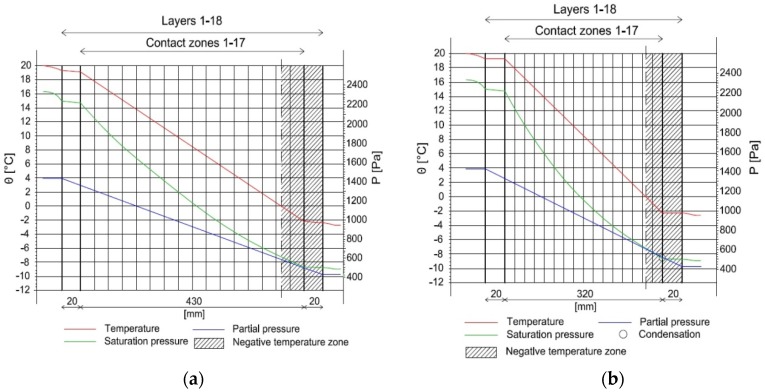
Distribution of temperature and water vapor pressure in (**a**) wall H1 and (**b**) wall FH1 for January.

The distribution of temperature, as well as pressure in both considered wall constructions is presented in Figure 12 for January, established as the critical month, characterized by the highest possibility of condensation.

**Figure 13:**
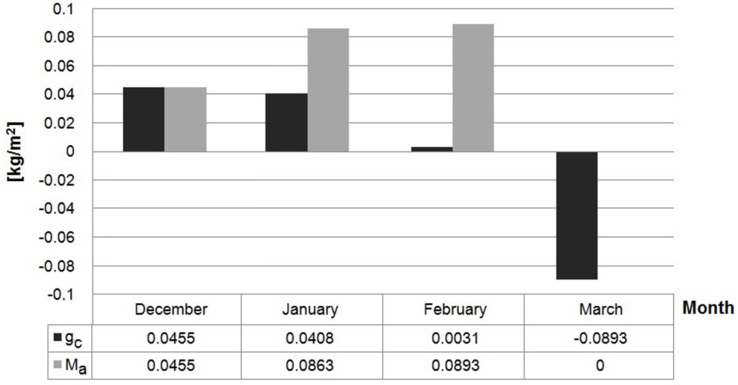
Streams of condensation (g_c_) and accumulated moisture (M_a_) in wall FH1 in the months in which condensation occurs.

The value of stream of condensation and the accumulated moisture in the considered wall are presented in Figure 13 for selected months.

## 4. Discussion

### 4.1. Physical Parameters of the Composites

#### 4.1.1. Density and Porosity

The utilized composites were characterized by the density ranging 2.05–2.22 g/cm^3^, and 1.89–2.19 g/cm^3^ for Series 1 and Series 2, respectively. Their apparent density was in the range of 356 to 476 kg/m^3^ and 405 to 467 kg/m^3^ depending on composition, which corresponds to literature data. Similar composites manufactured and examined by other authors were characterized by apparent densities ranging between 258 and 463 kg/m^3^ [43] and between 275 and 440 kg/m^3^ [44]. The apparent density of other plant origin composites, such as straw-clay composite, ranged 241–531 kg/m^3^ [45]. By contrast, the density of the another popular material used for energy saving constructions, namely autoclaved aerated concrete ranges from 300 to 700 kg/m^3^ depending on class, as stated in EN 678 standard.

According to authors’ own investigations and literature sources [43,46], density is determined by the ratio of binder to filler. Adding more binder to the mixture raises the weight of the composite, as does water. However, when there is too much water in the mixture, the latter becomes prone to stronger compaction, for instance, under the effect of gravity. Additionally, the weight ratio of binder/filler also had an impact on the apparent density of the composite, as noted in Series 1, where an increased amount of hemp (filler) decreased the density of the composite.

As far as Series 2 is concerned, the ratio of hemp shives and flax was modified, but the ratio of binder and filler remained constant. Dimensions of flax straws influence the composite density. Tiny fractions, due to their greater specific area, necessitate increasing the amount of binder in the mixture, thus enhancing the composite density. This information was also confirmed in the literature report [31], where the composites with segregated shives fractions were compared. The composite containing the smallest hemp-shives fractions was characterized by the greatest density.

**Figure 14:**
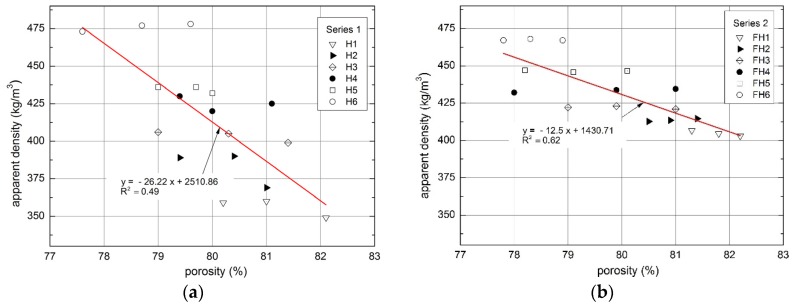
Dependences between composite porosity and apparent density: (**a**) Series 1; and (**b**) Series 2.

Low value of apparent density is the consequence of high porosity of the material. This may stem from a number of factors, including macropores (occurring due to the deficient arrangement of shives), mesopores (appearing between the shives and the binder), micropores (found between the hydrates in the binder matrix [14]), and the pores in the structure of the shives. Porosity of the investigated composites varies within 78.6–81.1% and 78.3–81.8% for Series 1 and 2, respectively. According to the literature reports [25], the porosity determined for the apparent density ranging from 258 to 463 kg/m^3^ reached the value between 72% and 84.9%; for the apparent density of 304 kg/m^3^, the porosity amounted to 80% [44], and for the apparent density of 478 kg/m^3^, it equaled 76.4% [47]. Similar dependences were achieved in our investigations. The relationship between the porosity of a composite and its apparent density is presented in Figure 14.

For the least dense composites (350–400 kg/m^3^), the porosity varies between 80% and 82%, whereas in the case of higher densities (450–470 kg/m^3^), the porosity is lower than 80%. It can be assumed that the relation between the apparent density and porosity is linear, described with regression functions presented in Figure 17. Coefficient of determination (R^2^) for Series 1 equals 0.49 and, for Series 2, it is 0.62. Those values are not high, but should be considered as typical for inhomogeneous composites manufactured on the basis of biological materials with variable fractions of shives and straws.

#### 4.1.2. Composites Absorptivity

Results of mass absorptivity presented in Figure 10 show only the maximum values in saturated states. It must by underlined that the water absorption process is very rapid and during the experiment, majority of absorbable water was taken in by the composite shortly (within several seconds) after they come into contact. The composites from Series 1 exhibited the mass absorptivity ranging from 93.7% to 162.8%. However, sample H1 failed the absorptivity test due to severe damage caused by water. This sample contained too much hemp fibers in relation to lime and cement. The composite absorptivity increases along with the filler content, which is correlated with the hydrophilic nature characterizing hemp shives. 

The mass absorptivity of composites belonging to Series 2 ranged 89.3–148%. However, the density is dependent on the ratio of filler components, namely hemp and flax shives. Adding flax shives raises the density of a composite and simultaneously lowers its absorptivity. It may result from fact that the smaller fractions are adjusting better during composition compaction. This influences the amount and size of the technological pores present between randomly dispersed shives, thus affecting the absorptivity value.

The ratio of flax and hemp shives was comparable in the case of both FH3 and FH4; however, the latter comprised more cement and less lime. This, in turn, raised the tightness and density of FH4, while lowering its absorptivity by 8.3%, in relation to FH3.

**Figure 15:**
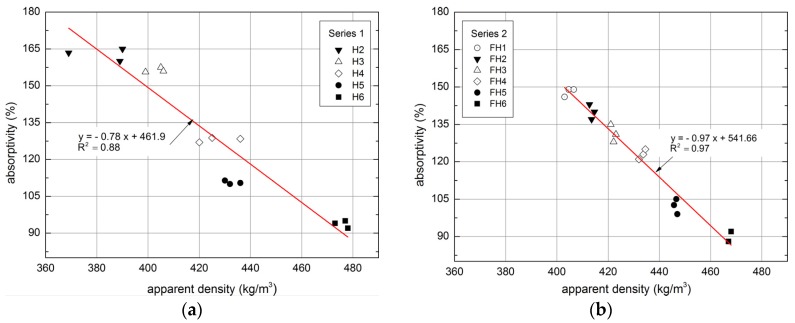
Relationship between absorptivity and apparent density of tested composites: (**a**) Series 1, (**b**) Series 2.

A relationship between the absorptivity of a composite and its apparent density can be observed (Figure 15).

The dependence between the apparent density and mass absorptivity can be described using linear regression functions. Coefficient of determination (R^2^) of regression formulas obtained for series 1 equals 0.88 and for series 2:0.97, which means that the applied model of regression covers about 90% of achieved data and more. In order to compare the obtained results and dependences with literature reports, it should be mentioned that, in certain studies, the absorptivity was lower by approximately 25% at a comparable density [18]. In order to achieve this value, the author employed a composite characterized by the apparent density of 1070 kg/m^3^, 4–8 mm shives fraction and the volume content of shives, binder and water amounting to 40%:29%:31%, respectively. In the cited research, the lowest absorptivity value was achieved in a composite containing flax shives with the smallest fraction (4–8 mm). Similar dependence was obtained in authors’ own research, in which the composites of Series 2, containing flax straws (smaller than hemp shives), were characterized by lower absorptivity comparing to the composites of Series 1.

Lime–hemp composites are not covered by any standard, but there are regulations (namely Straw Bale Construction Building Code, 2013 IRC Approval, Appendix R) concerning other plant origin material as straw bale, permitted for application as a material for external walls. It can be built in the walls provided that a suitable protection against rain waters is used, for example in the form of lime plasters and special architectonical elements (for example the eaves overhanging the face of the wall). In the case of the lime–hemp composite, it is also recommended to use the external cover, which is presented in the analysis of the condensation process section. It must be remembered that the mass absorptivity examined here simulates only the extreme conditions of exposition on constant water contact, which would be highly unlikely to occur under actual conditions.

#### 4.1.3. Thermal Conductivity Coefficient

**Figure 16:**
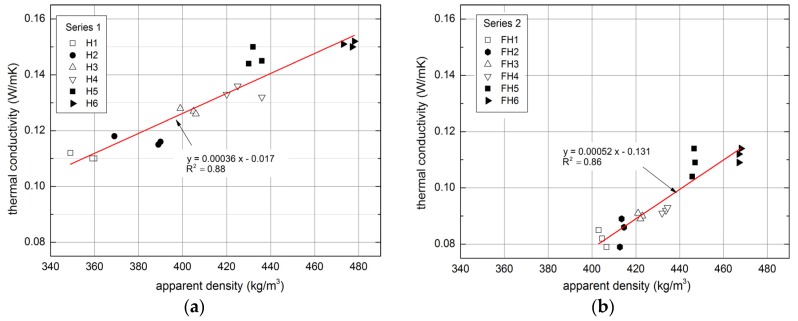
Relationship between apparent density and thermal conductivity of tested composites: (**a**) Series 1, (**b**) Series 2.

High porosity of the composite is conducive to its thermal insulation properties [43]. Figure 16 shows the relation between the apparent density of the composites and thermal conductivity coefficients.

The factors that influence the thermal conductivity coefficient λ of building materials involve density and humidity [48]. The dependence between the thermal conductivity and composite density that depends on binder/filler ratio, mixture placement, and compaction was determined in the conducted research. 

Thermal conductivity of the composite in Series 1 reached between 0.110 W/(m·K) and 0.151 W/(m·K). Thermal insulation was improved by high content of hemp shives in the composite. As indicated in the studies [39], although the thermal conductivity of a composite is affected by the amount of hemp filler, this relationship is non-linear. Fillers applied in Series 2 generated denser composites comparing to Series 1, which finally resulted in lower thermal conductivity values. This could be a consequence of different filler particles distribution characterizing both filler types. In addition, this generated greater compactness and homogeneity of Series 2 composites comparing to the Series 1.

The highest values of this parameter were found in the case of FH5 and FH6 composites—0.112 W/(m·K) and 0.109 W/(m·K), respectively. Both of these composites were characterized by the highest flax shives content and the highest apparent density. There is a visible tendency of the increase of thermal insulation features together with the increase of hemp shives – flax shives ratio in composition, even though the binder applied for all composites was of the same type.

The study conducted by Walker and Pavia proved that the thermal conductivity is influenced by the type of binder to a limited degree [37]. Moreover, according to Gourlay et al. [36], the composites comprising hydraulic lime exhibit greater thermal conductivity than the composites containing the hydrated lime, which is due to the different density of both binders. The structure formed by hydraulic lime is tighter and less porous in comparison to the one formed by hydrated lime. In turn, this results in higher density and lower thermal resistance.

Figure 16 presents the dependence between the apparent density of the composites and thermal conductivity coefficients as linear. In the case of Series 1, the goodness-of-fit for the applied model, expressed as coefficient of determination (R^2^) reaches 0.88 (see Figure 16) and in case of Series 2, R^2^ value equals 0.86.

Conducted research confirmed the dependence between material apparent density and thermal conductivity.

#### 4.1.4. Water Vapor Permeability Coefficient and Diffusion Resistance Factor

According to the results presented in Table 2, the type of binder, and its ratio to filler determine the water vapor permeability of the given material. The considered composites (H1 and FH1) yielded similar results (5.28 and 5.53 [-] respectively), which may be attributed to their comparable density (405 kg/m^3^ and 356 kg/m^3^). Walker et al. [49] analyzed a similar lime–hemp composites characterized by the density of 508 kg/m^3^ to 627 kg/m^3^ , and obtained comparable values of µ factor, ranging from 5.42 to 5.71 [-], which are comparable to the resistance factors achieved within the conducted experiment. 

### 4.2. Discussion on Mechanical Properties of the Examined Composites

#### 4.2.1. Compressive Strength of the Composites

Tested composites are characterized by certain mechanical properties that should enable transferring its own load plus side loads from wind. The application of this composite involves filling the load-bearing timber frame of the external walls, as well as transferring loads from both the roof and the floor. 

**Figure 17:**
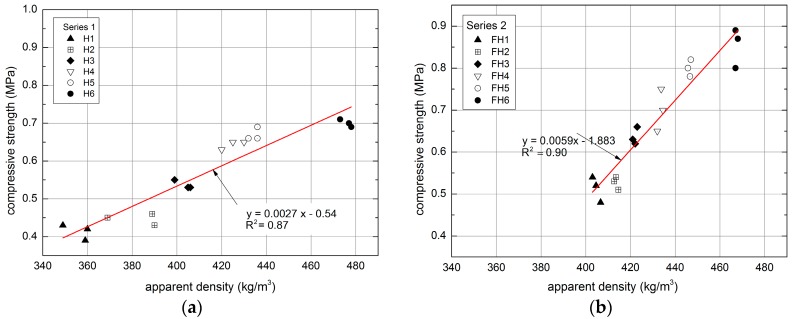
Relationship between apparent density and compressive strength of tested composites: (**a**) Series 1, (**b**) Series 2.

According to Table 3 and Figure 17, the compressive strength of composites ranges 0.41–0.70 MPa and 0.51–0.85 MPa for Series 1 and 2, respectively. This parameter is affected, for instance, by the type of binder, its ratio to the filler, the fraction of shives, as well as the method of mixture compaction [18,29,37,50]. In the case of Series 1, the compressive strength was influenced both by the varying proportions of hemp shives and the binder, as well as the changing ratio of Portland cement to the binder, used as a partial replacement for the hydrated lime. The compressive strength obtained for a composite characterized by the density of 404.73 kg/m^3^ equaled 0.41 MPa, with the ratio of filler to binder amounting to 0.9. On the other hand, H6 composite, boasting the density of 467.33 kg/m^3^, exhibited the compressive strength of 0.70 MPa, with the filler-binder weight ratio amounting to 0.3.

The highest amount of cement was used in H6 composite (highest strength in Series 1); conversely, the smallest amount of cement was used H1 composite (lowest strength in the whole experiment). In the case of Series 2, the compressive strength was determined by the type of the filler, with the highest strengths achieved by the composites characterized by the highest quantity of flax shives, namely FH6 (100% filler content by weight, highest strength), FH5 (70%, second highest strength), and FH4 (50%, third highest strength, equal to H6 from Series 1, with the highest amount of cement). Comparing the obtained values with the literature data, it was noticed that similar composite manufactured by Elfordy et al. [19] was composed of lime-based binder, hemp shives, and water in the amount of 34%:16%:50% by weight, respectively. Its compressive strength was in the range 0.18–0.85 MPa at densities of 290–610 kg/m^3^.

The examined composites can be compared to autoclaved aerated concrete (AAC) as a bearing material, but the strength of AAC with the density of 300 kg/m^3^ equals 1.5 MPa. According to EN 771-4 standard, the lowest compressive strength of the AAC should not be lower than 1 MPa. The greatest compressive strength of the examined composites equals 0.85 MPa; therefore, the tested composites cannot be used as bearing materials. However, they can serve as fillers of the external walls made as timber frame construction, mainly due to their good thermal properties.

**Figure 18:**
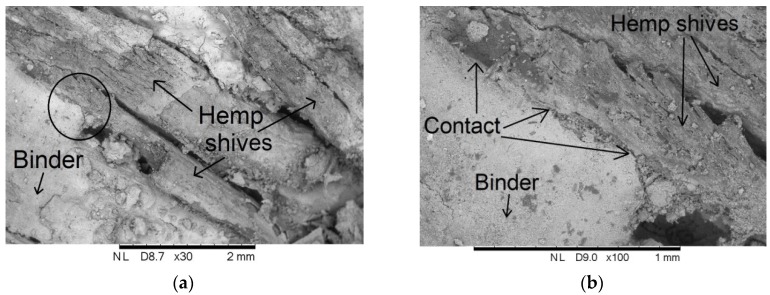
Scanning Electron Microscope (SEM) Image of contact between hemp shiv and binder of lime–hemp composite: (**a**) magnification 30×, and (**b**) magnification 100×.

According to literature data [14,15], the adhesion of binder to the shives has an influence on the behavior of composite under loading. At the initial phase of sample loading, the compressive strength is determined by the binder. The deformation of composite greatly increases after the connection between shives and the binder is broken [14]. Incorrect mixing or compacting of the samples may lower the effectiveness of the connection. In addition, the contact area between both materials can crack in the course of composite drying. This may be caused by the different characteristics of particular components and different volume changing processes during drying [15], which may weaken the contact area between the matrix and the filler. Figure 18 presents the binder-filler contact area of the random composite selected from Series 1. The picture in Figure 18 was taken by means of a scanning electron microscope.

#### 4.2.2. Flexural Strength

The flexural and compressive strengths of composites are affected by similar factors. Therefore, the parameters governing both increases and decreases resemble the ones discussed in the previous case. According to Table 3, the flexural strength characterizing the composites ranged 0.05–0.24 MPa and 0.09–0.24 MPa for Series 1 and Series 2, respectively. In relation to the compressive strength, the flexural strength increased to a lesser degree, for instance, the lowest value of flexural strength corresponds to approximately 21% of the highest obtained value. By contrast, the lowest value of compressive strength constitutes roughly 59% of the highest value.

**Figure 19:**
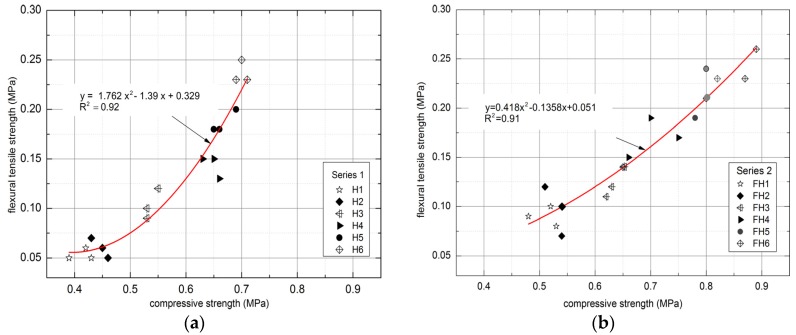
Relationship between compressive and flexural tensile strength of tested composites: (**a**) Series 1, (**b**) Series 2.

According to literature investigations, a dependence between the compressive and flexural strength of the materials is observed [51,52,53]. Figure 19 demonstrates the relation between both the compressive and the flexural tensile strength of the examined lime–hemp and lime–flax–hemp composites obtained within the described experiments.

In the course of the study, it was noted that the compressive and flexural strength correspond to each other. The polynomial trend exhibited high determination coefficient for Series 1 and 2, equaling R^2^ = 0.92 and R^2^ = 0.91, respectively. The ratios of binder/hemp and binder/flax–hemp had an influence on the obtained relations. In the case of Series 1, the highest results were achieved for H6 composite, which contained the lowest amount of hemp, namely 30%. By contrast, the highest results in Series 2 were observed for FH6 composite, which had the greatest content of flax, amounting to 70%.

Additionally, it should be mentioned that the strength of materials depends on the maturation time. The impact of maturation time on the flexural strength of a composite was investigated by Walker [34]. For the composites with the density of 508–627 kg/m^3^, the flexural strength after three months and after one year of maturation ranged 0.06–0.13 MPa and 0.1–0.2 MPa, respectively. Our strength results present values obtained after 28 days of maturation.

### 4.3. Discussion on Condensation Phenomenon

#### 4.3.1. Internal Surface Temperature to Avoid Critical Surface Humidity

As presented in Table 4 and mentioned in Section 3.3.1, the critical month for calculation is January, due to highest value of f_Rsi.min_ factor (f_Rsi.crit_), equal to 0.814. The value calculated for December is close to the critical value obtained for January and equals 0.811. Temperature coefficient f_Rsi_ calculated using the applied method amounted to 0.939. This value exceeds the critical ratio (0.814 for January), which prevents mold from growing on the internal surface in the course of an entire year.

#### 4.3.2. Determination of the Possibility of Interstitial Condensation

According to the graphical presentation in Figure 12, showing the distribution of temperature and water vapor pressure, condensation will not appear in the case of wall H1. As far as the wall made of FH1 composite (from Series 2) is concerned, condensation appears in the 17th contact zone, located between the 17th and 18th calculation layer, where two materials characterized by varying diffusion resistance come into contact. In January, the negative temperature was observed between the 16th and 17th contact zones on the external surface. December is characterized by the greatest total stream of condensation g_c_ amounting to 0.0455 kg/m^2^. On the other hand, the highest value of accumulated moisture, namely 0.0893 kg/m^2^ was found in February. Nevertheless, all the condensate that accumulated in the period of December to February should evaporate in March. 

In the winter period, the water vapor condensates at the contact point between the external plaster and composite layer due to low temperature and the differences between the water vapor permeability of these two materials. Lime plaster is characterized by higher diffusion resistance comparing to the examined composites H1 and FH1. This layer of plaster is necessary for composite protection against atmospheric factors, mainly rainfalls. Nevertheless, this difference is not significant, enabling the accumulated condensate to evaporate and, according to calculations presented in Section 3.3, it evaporates completely in March.

## 5. Conclusions

The research on the prospective application of flax and hemp acquired from Polish crops proved that both can be successfully utilized for the production of building materials. The characteristics of these materials are determined by the composition of the employed mixture. Flax and hemp, being natural products, are conducive to the sustainable development. As natural composites, they can be utilized—in conjunction with timber frames‑or the construction of fully recyclable buildings. 

A thorough analysis of the obtained results enables to formulate the following conclusions:

1. Biological composites based on hemp and flax are characterized by low strength parameters, which prevent them from being applied as bearing materials. The compressive strength of the tested composites was below 1 MPa, which makes them inferior to the lightest autoclaved aerated concretes.
2. Thermal properties of the tested composites are promising for the application as an insulating material. In the case of the lightest mixtures, thermal conductivity coefficient values are below 0.1 W/(m·K), which makes them superior to autoclaved aerated concretes.
3. Composites based on flax straw and hemp shives fillers exhibit better thermal properties than the composites containing only hemp shives as filler.
4. Good thermal properties and poor durability makes lime–hemp and lime–flax–hemp composites an appropriate filling material for timber frame constructions of the external walls, even without additional insulation.
5. Manufactured composites are characterized by low apparent density, ranging 356–476 kg/m^3^. This value is similar to lightweight autoclaved aerated concrete and should therefore ensure a relatively low weight of the constructed building.
6. The density of the composite and the related thermal conductivity are both determined by the content of flax and hemp fillers. The increase of filler content simultaneously lowers the apparent density and decreases thermal conductivity coefficient.
7. The composite produced from hemp and flax is characterized by high absorptivity, in most cases exceeding 100% by weight. It value may be lowered by the increase of binder content.
8. Lime–flax–hemp composite can be used for the construction of external walls, preventing the growth of mold and mitigating the negative impact of interstitial condensation that occurs in the wall to a limited degree, and can be quickly evaporated during the first spring days.
9. Lime–hemp composites, due to high water vapor permeability (μ = 5.28 and 5.53), are appropriate materials for the external walls, when using the suitable external and internal plasters providing diffusive openness of the whole barrier.

## Figures and Tables

**Table 1:** Proportions of compounds used for composites preparation (percentage by weight).

Material	Unit	Series 1						Series 2					
		H1	H2	H3	H4	H5	H6	FH1	FH2	FH3	FH4	FH5	FH6
Lime	(%)	23.8	20.8	21.0	21.3	22.2	23.8	18.9	18.9	18.9	11.8	18.9	18.9
Cement	(%)	2.5	3.9	4.6	5.3	5.6	5.2	6.2	6.2	6.2	11.8	6.2	6.2
Clay	(%)	-	-	-	-	-	-	1.9	1.9	1.9	2.3	1.9	1.9
Hemp shives	(%)	23.8	17.4	15.4	13.3	11.1	8.7	18.9	13.5	9.5	11.3	5.4	-
Flax straw	(%)	-	-	-	-	-	-	-	5.4	9.5	11.3	13.5	18.9
Sand	(%)	11.4	8.6	7.7	6.7	5.6	4.3	-	-	-	-	-	-
Water	(%)	39.5	49.3	51.2	53.3	55.6	58.0	54.1	54.1	54.1	51.6	54.1	54.1

**Table 2:** Coefficient of water vapor permeability (δ) and factor of diffusion resistance (µ) of composites (± SD).

Composite	Water Vapor Permeability Coefficient (kg/(m∙s∙Pa))	Water Vapor Diffusion Resistance Factor (-)
H1	3.79 × 10^−11^ ± 4.3 × 10^−13^	5.28 ± 0.06
FH1	3.62 × 10^−11^ ± 5.2 ×10^−13^	5.53 ± 0.08

**Table 3:** Mechanical properties of composites (average value ± SD).

Property	Series 1						Series 2					
	H1	H2	H3	H4	H5	H6	FH1	FH2	FH3	FH4	FH5	FH6
Compressive strength (MPa)	0.41 ± 0.023	0.45 ± 0.021	0.54 ± 0.027	0.65 ± 0.031	0.67 ± 0.029	0.70 ± 0.032	0.51 ± 0.028	0.53 ± 0.026	0.64 ± 0.029	0.70 ± 0.030	0.80 ± 0.032	0.85 ± 0.033
Flexural Strength (MPa)	0.05 ± 0.002	0.06 ± 0.002	0.1 ± 0.004	0.14 ± 0.005	0.19 ± 0.005	0.24 ± 0.007	0.09 ± 0.003	0.1 ± 0.002	0.13 ± 0.003	0.17 ± 0.005	0.21 ± 0.007	0.24 ± 0.008

**Table 4:** The results of the calculations.

Month	p_e_ (Pa)	Δp (Pa)	p_i_ (Pa)	p_sat(__θ__si,min)_ (Pa)	θ_si,min_ (°C)	f_Rsi,min_	f_Rsi_
I	428	915	1435	1794	15.8	0.814	0.939
II	449	887	1424	1780	15.7	0.803	
III	624	680	1373	1716	15.1	0.709	
IV	844	437	1326	1657	14.6	0.496	
V	1205	227	1454	1818	16.0	0.287	
VI	1430	154	1599	1999	17.5	0.343	
VII	1495	126	1633	2041	17.8	0.301	
VIII	1487	126	1625	2032	17.8	0.277	
IX	1221	292	1542	1927	16.9	0.572	
X	928	466	1440	1800	15.8	0.639	
XI	598	757	1431	1789	15.8	0.773	
XII	453	895	1438	1797	15.8	0.811	
